# Association between dietary patterns and bacterial vaginosis: a case–control study

**DOI:** 10.1038/s41598-022-16505-8

**Published:** 2022-07-16

**Authors:** Morvarid Noormohammadi, Ghazaleh Eslamian, Seyyedeh Neda Kazemi, Bahram Rashidkhani

**Affiliations:** 1 Department of Cellular and Molecular Nutrition, Faculty of Nutrition and Food Technology, National Nutrition and Food Technology Research Institute, Shahid Beheshti University of Medical Sciences, No 7, Hafezi St., Farahzadi Blvd., P.O.Box: 19395-4741, Tehran, 1981619573 Iran; 2 Department of Obstetrics and Gynecology, School of Medicine, Imam Hossein Hospital, Shahid Beheshti University of Medical Sciences, Tehran, Iran; 3 Department of Community Nutrition, Faculty of Nutrition and Food Technology, National Nutrition and Food Technology Research Institute, Shahid Beheshti University of Medical Sciences, Tehran, Iran

**Keywords:** Epidemiology, Diseases, Health care, Medical research, Risk factors

## Abstract

Bacterial vaginosis (BV) is a predominant vaginal disturbance that affects about 25% of childbearing-aged women. Dietary consumption may have a crucial role in vaginal flora imbalances. This study was a hospital-based case–control study. In total, 144 incident BV cases and 151 healthy participants were recruited from the gynecology clinic in Tehran, Iran, between November 2020 and June 2021. Participants' typical diets were collected by a food frequency questionnaire. Vaginal flora was characterized based on the Amsel criteria. Factor analysis was used to pinpoint the principal dietary patterns. For logistic regression, the first tertile was assumed as a reference. Five principal dietary patterns emerged and were nominated as "Healthy diet," "Unhealthy diet," "Ovo-vegetarian diet," "Pseudo-Mediterranean diet," and "Western diet." The "Unhealthy diet" pattern were positively associated with BV (adjusted odds ratio (aOR) = 3.35; 95% confidence interval (CI) 1.41, 7.94; p_trend_: 0.006), while adherence to the "Ovo-vegetarian diet" pattern was associated with a reduced odds of BV (aOR = 0.16; 95% CI 0.07, 0.34; p_trend_ < 0.001). These results provide evidence that following the "unhealthy diet" pattern may lead to developing BV, and plant-based eating patterns may be associated with reduced BV odds.

## Introduction

Bacterial vaginosis (BV), also called vaginal bacteriosis, is the principal reason for vaginal discharge, irritation, and odor in childbearing-aged women^1,2^. However, in most cases (about 50%), BV is often asymptomatic^3^. The global estimated prevalence of BV ranges from 23 to 29%^4^. In non-pregnant and pregnant Iranian women, BV prevalence is 28% and 16%, respectively^5^. A dysbiosis of the vaginal flora from the dominant *Lactobacillus* spp. to a mix of *G. vaginalis*, *Bacteroides* spp., *Mobiluncus* spp., and *Mycoplasma hominis*, characterizes BV^6^. BV may persist after treatment, and symptoms return in 50% of women within 12 months^7^. BV infection promotes the increased chance of pelvic inflammatory disorder, preterm childbirth, postpartum endometritis, and increased risk of other infections like Trichomonas vaginalis, Chlamydia trachomatis, and Vulvovaginal candidiasis^8^. The specific cause of BV is not clear yet, but several risk factors have been characterized, including smoking, vaginal douching, recent antibiotics usage, using intrauterine devices, and frequent sexual contacts^9^. Despite the adverse reproductive health outcomes of BV, there is little information about the relation between BV and dietary factors.

Vaginal flora imbalances may occur as a result of dietary intakes. Previous studies have suggested a potential relationship between BV and nutrients^10–14^. In Neggers et al. study, dietary fat was connected with an increased risk of BV^11^. According to Thoma et al., the dietary glycemic load was correlated with BV development and persistence^10^. Dietary intake is a multidimensional exposure. When it comes to addressing the multicollinearity of foods and understanding the relationship of dietary intakes to chronic disease, researchers increasingly look at the diet as a whole^15^. Therefore, using factor analysis to determine dietary patterns is a functional dietary assessment approach^16^. The 2015 Dietary Guidelines Advisory Committee defined dietary patterns as follows: "the quantities, proportions, variety or combination of different foods and drinks in diets and the frequency with which they are consumed"^17^.

To our knowledge, no earlier observational research has examined the association between dietary patterns and BV. Since considering foods alone does not show the synergistic effects of multiple diet components^18^, this research intended to examine the link between dietary patterns and BV.

## Methods

### Ethical considerations

This hospital-based case–control study recruited 148 women with bacterial vaginosis as cases and 153 healthy women from November 2020 until June 2021. The study was reviewed and authorized by the National Nutrition and Food Technology Research Institute, Shahid Beheshti University of Medical Sciences, Tehran, Iran. Participants signed the informed consent before inclusion. All procedures were conducted according to the latest version of the Helsinki Declaration (The ethics committee code: IR.SBMU.NNFTRI.REC.1399.054).

### Sample size calculation

According to the primary objective, dietary pattern exposure was used to compute the required sample size. The available data indicated that 73% of Iranian adults had adherence to unhealthy dietary patterns^19^. The odds ratio (OR) for BV was postulated 2.5 among women with unhealthy dietary patterns relative to non-adherence. A total of 125 BV-affected women (cases) and 125 healthy women (controls) were calculated to attain 80% statistical power for such effect size at a 5% level of significance^20^. To compensate for over- and underestimation of energy intake or withdrawal, 316 participants were recruited.

### Study population

Eligible participants were selected by convenience sampling method among the women referred to the gynecology clinic at Imam Hossein Hospital in Tehran, Iran. A gynecologist examined all patients to evaluate for bacterial vaginosis. Incident cases were diagnosed with a first BV according to the Amsel criteria with at least three of the four determining criteria: a homogeneous and dilute vaginal discharge, vaginal pH greater than 4.5, fish odor after adding 10% potassium hydroxide to the discharge slide, and the presence of 20% of clue cells under saline microscopy^21^.

Eligible participants were 18–45 years old, non-lactating, non-pregnant, not menopause and not suffering from systemic immunity diseases, chronic infection, chronic diet-related diseases (cancer, diabetes, cardiovascular disease, etc.), or any disease in the uterine cavity such as polyp and fibroids as well as lack of hysterectomy. Also, they did not consume vaginal douches, antibiotics, hormonal contraceptives, probiotics, and immunosuppressive drugs. Participants were included in the control group upon the absence of ongoing or previous BV. The exclusion criteria for both BV-affected and healthy women included those who had reported energy intakes outside of the range of ± 3 standard deviation (SD) from the average energy intakes of the study population and were unable to respond to the questions.

### Socio-demographic assessment

A data collection form was developed to gather data on age, history of medication and supplementation, BV family history, education level, occupational status, smoking, number of sex partners, and monthly family income. Due to religious and cultural beliefs in Iranian society, questions regarding alcohol and opium were not determined.

### Non-dietary exposure assessment

Anthropometric measures, including weight, height, and waist circumference (WC) were performed following standard protocol by the one qualified examiner for all participants to avoid random observer error. Bodyweight (in kg to the nearest 100 g) was measured with a reliable scale while participants wore light clothing and without shoes. To assess central adiposity, WC (in cm to the nearest 0.1 cm) was measured by an unstretched tape measure at the umbilical site, at the abdominal level, on light clothing, in a standing position, without any pressure on the body surface. Body height (in cm to the nearest 1 mm) was measured by standing and straight tape. The participants were asked to stand straight while the shoulders were in a normal position, without shoes, and with the heels together. Next, the body mass index (BMI) was computed by dividing weight by height squared (square meters). BMI and WC were categorized according to World Health Organization cut-off criteria^22^ and the first report of the Iranian National Committee on Obesity^23^, respectively. Physical activity assessment was based on the International Physical Activity Questionnaire (IPAQ), and its validity and stability have been examined in a previous study in Iran^24^.

### Dietary intake assessment

Individuals' typical diets were gathered with a valid and reliable semi-quantitative food frequency questionnaire (FFQ) developed and validated in the Iranian population^25^. Participants were asked about their usual intakes over the past year before diagnosing BV for cases and the previous year before the interview in controls. This FFQ has been designed according to the Willet method^18^, including 168 food items with a standard serving size for each food item. The average size of each food item was explained to the participants in the interview. Then, they reported the frequency of consumption of each food item given serving on a daily, weekly, or monthly basis. The mentioned values of each food were converted to grams using the household scale guide. The mean daily intakes of energy and nutrients were determined using the Iranian food composition table^26^ and the USDA food composition table^27^.

### Statistical analysis

Data were analyzed using IBM SPSS Software version 20.0 (SPSS, Inc.). All hypothesis tests were 2-tailed, with a P value less than 0.05 considered significant. Normal distribution of continuous variables was checked using Q–Q plots, histogram charts, and Kolmogorov–Smirnov test. General quantitative and categorical characteristics were expressed as median (interquartile range (IQR)) and frequency and percentages. A chi-squared test analyzed differences in the distribution of categorical variables (e.g., familial history of BV). Mann–Whitney test was applied to check differences in the distribution of continuous variables (e.g., physical activity). Factor analysis with the principal component analysis (PCA) method was used to simplify the factor structure to extract dietary patterns. FFQ Food items were classified into 30 groups based on similarity in nutrients, shown in Table 1. Kaiser–Meyer–Olkin (KMO = 0.634) and Bartlett's tests (P < 0.001) were used to endorse the sample size adequacy and data suitability for factor analysis. According to eigenvalue greater than 1.6, the scree plot and percentage of variance greater than 5%, factors (dietary patterns) were retained. Varimax rotation (orthogonal) was used to examine the relation between variables and factors. Food groups with a factor loading of more than 0.4 remained, and factors were specified based on them. Factor scores were measured for each participant, and they were categorized into tertiles. The first tertile was assumed as a reference. The base and multivariable-adjusted odds ratios (OR) with 95% confidence intervals (CIs) were determined by the logistic regression to expound the association between dietary patterns and odds of BV. Adjusted ORs were computed by adjusting for familial history of BV, BMI (Kg/m^2^), WC (cm), cigarette smoking, energy intake (Kcal/d), calcium supplement, and physical activity (MET/h/d).

**Table 1:** Food groups and food items of FFQ used in the analysis.

Food groups	Food items
Refined grains	Iranian bread (Lavash, taftoon), baguette bread, toast, rice, pasta, vermicelli, flour
Whole grains	Iranian bread (Barbari, Sangak)
Fried potato	Fried Potato
Sweets and desserts	Biscuits, crackers, cake yazdi, homemade cakes, other types of cakes, dry sweets, fresh sweets, gaz, sohan, chocolate, caramel cream, homemade halva, sugar halva, noghl, donuts, jams
Beans	Lentils, beans, peas, mung beans, chickpeas
Red meat	Beef, mutton, ground meat
Processed meat	Sausages, burgers, salami
Visceral meat	Liver, heart, kidney, pache, sirabi
Fish	Fish, tuna fish
Poultry	Poultry
Egg	Egg
High fat dairy	High-fat milk, high-fat yogurt, greek yogurt, cream cheese, cream, traditional ice cream, non-traditional ice cream, kashk
Low fat dairy	Low fat yogurt, low fat milk, cheese, dough
Yellow vegetables	Carrot, pumpkin
Starchy vegetables	Green peas, beet root, corn, broad beans, potato
Green vegetables	Spinach, lettuce, cucumber, squash, green peppers, cabbage, celery, green beans, bell peppers
Other vegetables	Garlic, onion, eggplant, tomato, mushrooms
Vegetable oil	Margarine, vegetable oils in liquid form (except for olive oil)
Solid Oils	Tallow, solid oil, animal oil, butter
Olive and olive oil	Olive and olive oil
Red fruits	Watermelon, cherry, berries, pomegranate, strawberry
Yellow fruits	Melon, cantaloupe, pear, apricot, peach, nectarine, grapefruit, orange, banana, sweet lemon, tangerine, persimmon
Other fruits	Apples, figs, green tomatoes, grapes, kiwi, dates, plums
Sweet drinks	Sweet soft drinks
Sugar	Sugar, cube sugar, candy, honey
Snacks	Potato chips, puff
Nuts	Almonds, walnuts, peanuts, hazelnuts, pistachios, and seeds
Pickles	Pickles
Dried fruits	Raisins, Dried Apricots, Dried Peaches, Dried Figs, Dried Berries
Fruit juice	Carrot juice, orange juice, melon juice

### Ethics approval

The study was reviewed and approved by the National Nutrition and Food Technology Research Institute, Shahid Beheshti University of Medical Sciences, Tehran, Iran. The signed informed consent form was obtained from each participant before inclusion. All procedures were conducted according to the latest version of the Helsinki Declaration. The ethics committee code was IR.SBMU.NNFTRI.REC.1399.054.

## Results

**Figure 1:**
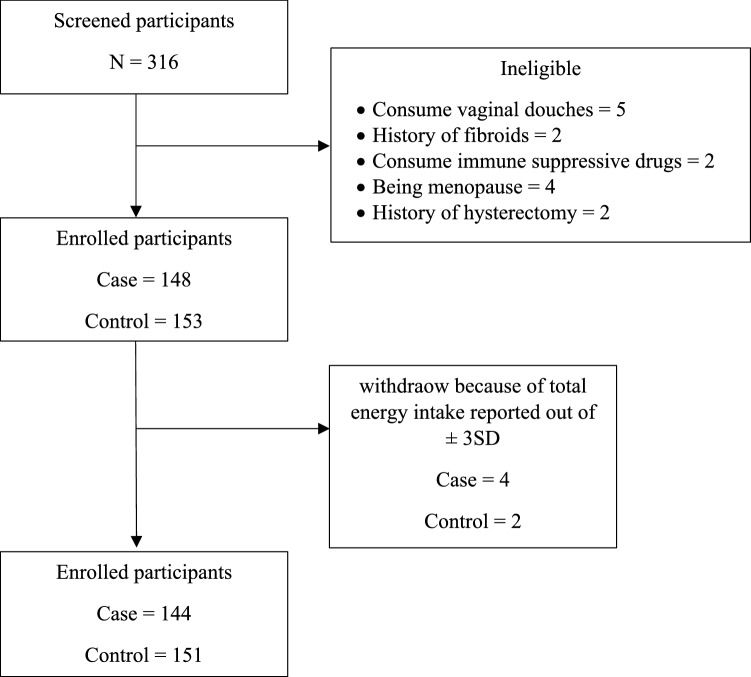
Study flow diagram.

After estimating the participants' calorie intake, 4 BV-affected women and 2 healthy women whose log scale of total energy intake was either >  + 3SD or < -3SD from the mean were excluded from the mean statistical analysis (Fig. 1). Therefore, participation rates were 97.3% and 98.7% among BV-affected and healthy women, respectively.

Baseline characteristics of the BV-affected women (n = 144) and healthy women (n = 151) are demonstrated in Table 2. The median (IQR) age of participants was 30 (25–33) and 32 (24–37) years in BV-affected and healthy women, respectively. Compared to the healthy women, BV-affected women tended to have higher levels of obesity (P = 0.016), higher levels of abdominal obesity (P = 0.05), higher frequency of familial BV (P < 0.001), higher smoker (P < 0.001), and lower calcium supplement use (P = 0.016). There was no statistically significant difference concerning other general characteristics between BV-affected and healthy women.

**Table 2:** Baseline characteristics of participants in case and control groups.

Characteristic*	BV-affected women n = 144	Healthy women n = 151	P Value^†^
Age, year, median (Q_1_–Q_3_)	30 (25–33)	32 (24–37)	0.177
Familial history of BV	77 (53.5)	37 (24.5)	< 0.001
**Education**			0.408
Primary/secondary school	37 (25.7)	39 (25.8)	
Bachelor’s degree	76 (52.8)	70 (46.4)	
Master’s/Doctoral degree	31 (21.5)	42 (27.8)	
**Cigarette per day**			< 0.001
0	118 (82)	149 (98.7)	
1–2	12 (8.3)	2 (1.3)	
≥ 3	14 (9.7)	0 (0)	
Employment status, Employed	43 (29.9)	42 (27.8)	0.698
Monthly family income, < 250 US $	111 (77.1)	121 (80.1)	0.523
**Frequency of pregnancy**			0.885
0	64 (44.4)	70 (46.4)	
1–2	66 (45.8)	65 (43)	
≥ 3	14 (9.7)	16 (10.6)	
Menstrual cycle, Regular	95 (66)	102 (67.5)	0.774
**Number of sexual partners in the previous month**			0.794
0	43 (29.6)	44 (28.9)	
1	95 (66)	103 (68.1)	
≥ 2	6 (4.4)	4 (3)	
Physical activity (MET/h/day), median (Q_1_–Q_3_)	40.3 (35.2–44.7)	40.3 (36.4–46.6)	0.599
**BMI status**			0.016
Underweight and healthy	59 (41)	83 (55)	
Overweight	64 (44.4)	44 (29)	
Obese	21 (14.6)	24 (16)	
Abdominal obesity	50 (34.7)	37 (24.5)	0.05
Calcium supplement 500 mg/day	10 (6.9)	24 (15.9)	0.016
Folate supplement 400 μg/day	30 (20.8)	22 (14.6)	0.158
Vitamin D supplement 50,000 IU/month	49 (34)	66 (43.7)	0.088
Iron supplenet 30 mg/day	26 (18.1)	41 (27.2)	0.062

*BV* bacterial vaginosis, *BMI* body mass index.

*Values are No (%) unless otherwise noted.

^†^Using Mann–Whitney U or χ^2^ test/Fisher’s excact test, as appropriate.

Table 3 shows factor loading values of food groups for dietary patterns. Five principal dietary patterns were derived, representing 38.7% of the total variance. A "Healthy diet" pattern, represented 9.9% of the total variance, comprised high fruits (all kinds), poultry, and other vegetables consumption (Garlic, Onion, Eggplant, Tomato, Mushrooms). An "Unhealthy diet" pattern represented 8.6% of the variance and comprised sugar, solid oils, sweets and desserts, red meat, fried potato, refined grains, visceral meat, and sweet drinks. An "Ovo-vegetarian diet" pattern represented 8.4% of the variance, comprised all kinds of vegetables, beans, whole grains, and eggs. A "Pseudo-​Mediterranean diet" pattern represented 6.3% of the variance, comprised nuts, fish, olive, and olive oil. A "Western diet" pattern represented the slightest variance (5.6%) and comprised high intakes of processed meat, snacks, and pickles.

**Table 3:** Factor loading values of food groups for dietary patterns.

	Dietary patterns				
	Healthy diet	Unhealthy diet	Ovo-vegetarian diet	Pseudo-Mediterranean diet	Western diet
Red fruits	0.886				
Yellow fruits	0.873				
Other fruits	0.703				
Poultry	0.552				
Other vegetables	0.538		0.536		
Sugar		0.600			
Solid oils		0.585			
Sweets and desserts		0.562			
Red meat		0.526			
Fried potato		0.494		− 0.433	
Refined grains		0.456			
Visceral meat		0.450			
Sweet drinks		0.416			
High fat dairy					
Green vegetables			0.649		
Yellow vegetables			0.575		
Beans			0.571		
Starchy vegetables			0.474		
Egg			0.439		
Whole grains			0.406		
Low fat dairy					
Nuts				0.702	
Olive and olive oil				0.679	
Fish				0.454	
Vegetable oil					
Dried fruits					
Fruit juice					
Processed meat					0.651
Snacks					0.645
Pickles					0.640
Total Variance	9.91%	8.57%	8.35%	6.27%	5.63%

Factor loading values < 0.4 were excluded.

Table 4 presents the base and adjusted OR for odds of BV through the emerged dietary patterns' tertiles. In the base model, being in the last tertile of the "Pseudo-Mediterranean diet" was negatively associated with BV odds (OR = 0.53; 95%CI: 0.29, 0.95; P_trend_: 0.042). However, the association was not statistically significant after adjustment for potential confounders. In the full model, after controlling for the confounders, women in the second and the third tertiles of the "unhealthy diet" pattern had a 2.04 (95% CI: 1.02, 4.09; P_trend_: 0.04) and 3.35 (95%CI: 1.41, 7.94; P_trend_: 0.006) times, respectively, a higher chance for BV in comparison to women in the first tertile. The chance to experience BV was 84% (95%CI: 0.07, 0.34; P_trend_ < 0.001) lower in the third tertiles of the "Ovo-vegetarian diet" pattern, in comparison to women in the first tertile. There was no significant relationship between BV odds and other dietary patterns.

**Table 4:** Association between tertiles of dietary patterns and odds of bacterial vaginosis among the participant.

Dietary patterns	Tertiles of dietary patterns			P_Trend_
	1st	2nd	3rd	
**Healthy diet**				
No. cases/no. controls	53/50	57/51	33/50	
Base model^†^	1.00 (Ref.)	1.05 (0.61, 1.81)	0.62 (0.35, 1.12)	0.131
Full model^‡^	1.00 (Ref.)	0.82 (0.44, 1.53)	0.59 (0.30, 1.17)	0.132
**Unhealthy diet**				
No. cases/no. controls	29/50	43/51	71/50	
Base model^†^	1.00 (Ref.)	1.45 (0.79, 2.68)	2.45 (1.37, 4.39)	0.002
Full model^‡^	1.00 (Ref.)	2.04 (1.02, 4.09)	3.35 (1.41, 7.94)	0.006
**Ovo-vegetarian diet**				
No. cases/no. controls	75/50	53/51	15/50	
Base model^†^	1.00 (Ref.)	0.69 (0.41, 1.17)	0.20 (0.10, 0.39)	< 0.001
Full model^‡^	1.00 (Ref.)	0.57 (0.31, 1.04)	0.16 (0.07, 0.34)	< 0.001
**Pseudo-Mediterranean diet**				
No. cases/no. controls	57/50	56/51	30/50	
Base model^†^	1.00 (Ref.)	0.96 (0.56, 1.65)	0.53 (0.29, 0.95)	0.042
Full model^‡^	1.00 (Ref.)	1.08 (0.59, 1.97)	0.61 (0.31, 1.19)	0.187
**Western diet**				
No. Cases/no. controls	48/50	42/51	53/50	
Base model^†^	1.00 (Ref.)	0.86 (0.49, 1.52)	1.10 (0.64, 1.92)	0.717
Full model^‡^	1.00 (Ref.)	0.73 (0.39, 1.36)	0.80 (0.43, 1.51)	0.484

Logistic regression model.

^†^Base OR.

^‡^Adjusted for familial history of BV, BMI (Kg/m^2^), WC (cm), cigarette smoking, energy intake (Kcal/day), calcium supplement use and physical activity (MET/h/day).

## Discussion

This is the first case–control study to assess the association between dietary patterns and BV to the best of our knowledge. In this research, five principal dietary patterns, including "Healthy diet," "Unhealthy diet," "Ovo-vegetarian diet," "Pseudo-Mediterranean diet," and "Western diet," were identified. Among the dietary patterns obtained, the "Unhealthy diet" pattern, defined by high loading of sugar, solid oils, sweets and desserts, red meat, fried potato, refined grains, visceral meat, and sweet drinks, was substantially associated with a higher BV odds. In contrast, a considerable association was found between the "Ovo-vegetarian diet" pattern high in all kinds of vegetables, beans, whole grains, and egg and BV in the multivariable-adjusted model. In addition, women with the highest adherence to the "Pseudo-Mediterranean diet" pattern high in nuts, and fish, olive, and olive oil also had lower odds for BV in the base model. No clear association was found in relation to the "Healthy diet" and the "Western diet."

The main characteristic of the "Unhealthy diet" pattern is a higher intake of saturated fat sources such as solid oils, red meat, fried potato, visceral meat. Similar to our finding, Neggers et al. have shown that saturated fat increases BV risk^11^. Although the mechanism for the role of saturated fat consumption in BV occurrence remains unknown, a high saturated fat consumption may lead to an elevated vaginal pH and alter the vaginal microflora^3,11^. Moreover, a high-saturated fat diet may also affect the mucosal immune system^11^. Another main characteristic of the "Unhealthy diet" pattern is high dietary carbohydrate content with a high glycemic index and glycemic load, including sugar, sweets, desserts, refined grains, and sweet drinks. Consistent with our finding, Thoma et al. have demonstrated a direct link between dietary glycemic load and BV progression and persistence^10^. High-GI/GL foods overconsumption as an unhealthy diet may be related to the pathogenesis of BV and could influence host response to bacterial colonization through oxidative damage and impaired immune response^28,29^. In return, participants adhering to a diet composed mainly of plant-based foods had a lower likelihood of BV in our study. The main components of the "Ovo-vegetarian diet" pattern include high-fiber and starchy foods, including vegetables, beans, and whole grains. In support, according to Shivakoti et al., higher fiber intake was inversely associated with the odds of molecular-BV^30^. Diets rich in fiber may reduce the risk of bacterial infections related to BV by affecting the microflora through more *Lactobacillus*-dominant profiles and positively impacting vaginal health^30^. Also, three previous studies showed a higher prevalence of BV among non-vegetarians than vegetarians, in line with our results^3,31,32^. The practical consequence of the "Ovo-vegetarian diet" pattern on BV can explain by creating a suitable environment for *Lactobacillus*^33^, lowering vaginal pH, and decreasing oxidative stress^34^. The vaginal micro ecological environment is affected by diets rich in starch due to high glycogen levels in the genital fluid^35^. Neggers et al. have also demonstrated an inverse association between severe BV and folate intakes^11^. Eggs, vegetables, and whole grains have been identified to represent the primary sources of folate. This result reveals the possibility that high intakes of folate sources may improve the immune system and are associated with decreased risk of BV^11^.

The current study has several potencies. The dietary pattern-based investigation was used to assess the total dietary intakes rather than individual dietary intakes. Since some foods may act synergistically, the dietary pattern approach provided better information about the correlation between dietary intake and diet-related diseases^36^. Dietary patterns derived from a validated-FFQ. Participants who under- or over-report their energy intakes were excluded. Newly diagnosed BV patients were included. Incident case selection would support a causal interpretation and control the recall bias^37,38^. High participation rates were obtained in both cases and controls. Information on several potential covariates was available to adjust in regression models. One person in the hospital laboratory performed a diagnostic test in both groups to control information bias. Besides, a trained dietician completed the questionnaires and was blinded to the diagnostic results during the interview. However, the current study has several limitations. This study did not use the Nugent score for BV diagnosis, although it is the gold standard for BV assessment. Although researchers tried to minimize bias as much as possible, selection bias, measurement bias, and recall bias might result in misleading findings in a case–control design. Different types of bacteria causing the BV were not considered in the current study. The cultural and religious taboo on alcohol and opium in Iran prevented the collection of data on these variables. Besides, the current study did not consider percentages of body fat, and the only criteria to assess obesity was the body mass index.

## Conclusion

To conclude, the results revealed that the "Ovo-vegetarian diet" characterized by all kinds of vegetables, beans, whole grains, and eggs decreases the odds of BV in this sample of Iranian women. However, the "Unhealthy diet" pattern defined by high sugar, solid oils, sweets and desserts, red meat, fried potato, refined grains, visceral meat, and sweet drinks increases the odds. Therefore, it is recommended that these findings should be considered in nutritional education and dietary recommendation to prevent BV. The findings need to be confirmed by further longitudinal dietary research to clarify whether dietary change is feasible and affects BV outcomes.

## Data Availability

Upon a reasonable request, the corresponding author will provide the data that support the findings of this research.
